# Partly intraoperative cell salvage in pediatric craniocerebral trauma: effects on coagulation function, allogeneic blood requirements, and clinical outcomes — a propensity score-matched retrospective cohort study

**DOI:** 10.3389/fped.2026.1825996

**Published:** 2026-05-26

**Authors:** Chao Wang, Lijing Li, Zhengzheng Gao, Jing Hu, Fang Wang

**Affiliations:** Department of Anesthesiology, Beijing Children’s Hospital, Capital Medical University, National Center for Children’s Health, Beijing, China

**Keywords:** autologous blood transfusion, coagulation function, intraoperative cell salvage, pediatric neurosurgery, propensity score matching, traumatic brain injury

## Abstract

**Background:**

Traumatic brain injury (TBI) in children frequently necessitates intraoperative blood transfusion owing to the hemorrhagic risk associated with hematoma evacuation. Intraoperative cell salvage (ICS) offers a strategy to reduce allogeneic blood exposure. However, the impact of partial usage of ICS on coagulation function in the pediatric neurosurgical setting remains insufficiently characterized.

**Methods:**

This retrospective cohort study enrolled 136 children (aged 1–18 years) who underwent cerebral hematoma evacuation between February 2016 and April 2022. Patients receiving combined autologous and allogeneic transfusion constituted the ICS group (*n* = 42), while those receiving allogeneic transfusion alone constituted the ABL group (*n* = 94). Propensity score matching (PSM) at a 1:1 ratio yielded 41 matched pairs. Perioperative coagulation indices (PT, APTT, INR, FIB, PLT), serum electrolytes, hemoglobin (Hb), hematocrit (Hct), and clinical outcomes were compared between groups before and after PSM.

**Results:**

Following PSM, allogeneic blood consumption was significantly higher in the ABL group (P < 0.001), while total red cell volume did not differ. No significant intergroup differences were detected in postoperative PT, APTT, INR, PLT, Hb, Hct, or serum electrolytes (all *P* > 0.05). The composite coagulation disorder rate was significantly lower in the ICS group both before PSM (19.0% vs. 44.7%; *P* = 0.008) and after PSM (17.1% vs. 39.0%; *P* = 0.049). Hypofibrinogenemia (FIB < 1.5 g/L) was significantly less frequent in the ICS group before and after PSM (*P* = 0.014 and *P* = 0.040, respectively). Clinical outcomes including operating time, ICU admission, intubation duration, and hospital stay did not differ significantly after PSM.

**Conclusion:**

Partial intraoperative autologous blood salvage in pediatric hematoma evacuation reduces allogeneic blood consumption and the incidence of postoperative coagulation disorders without impairing standard coagulation indices or electrolyte balance. These findings are hypothesis-generating and require confirmation in prospective multicenter trials.

## Introduction

Traumatic brain injury (TBI) remains a leading cause of morbidity and mortality in the pediatric population worldwide. Acute traumatic coagulopathy is commonly associated with TBI and is almost uniformly linked to worse clinical outcomes (1). In Asia, the incidence of TBI has been reported at approximately 380 per 100,000 person-years (2), and the population-based TBI mortality in China approximates 13 per 100,000 people, consistent with internationally reported rates (3).

**Figure 1:**
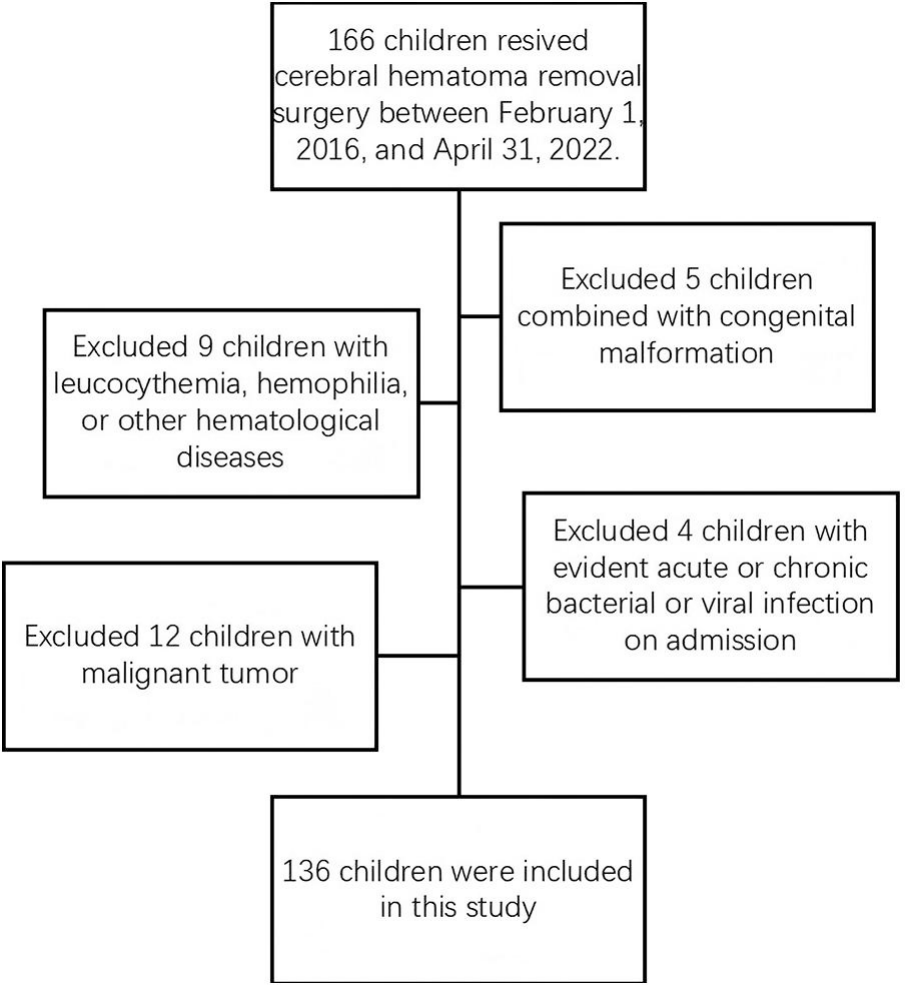
Flowchart of patient selection.

Craniotomy for cerebral hematoma evacuation carries substantial intraoperative hemorrhagic risk, and blood transfusion is frequently required to maintain hemodynamic stability in children undergoing this procedure (4). Despite evolving blood donation policies, global unmet demand for blood products persists (5). Allogeneic blood transfusion is associated with a spectrum of adverse outcomes, including transfusion-transmitted infections, incompatibility reactions, and transfusion-related immunomodulation (6) (Figure 1).

Intraoperative cell salvage (ICS) is a blood conservation technique whereby shed blood is collected, processed by centrifugal washing, and reinfused as packed autologous red blood cells (RBCs). Because this process removes plasma proteins, platelets, and clotting factors, concerns have been raised that ICS may adversely affect coagulation (7). However, clinical data in obstetric and other surgical populations have not demonstrated significant hemostatic deterioration following autologous reinfusion (8). ICS is increasingly adopted in adult neurosurgery (9, 10), yet robust pediatric-specific data from cranial trauma surgery are limited.

In the pediatric trauma setting, the most common intraoperative scenarios are allogeneic transfusion alone or combined ICS with supplemental allogeneic transfusion when autologous yield is insufficient. Comparing these two clinically representative groups directly addresses the question of whether partial ICS confers measurable hemostatic or clinical benefit. The primary objective of this study was to determine whether partial intraoperative autologous blood reinfusion alters postoperative coagulation function compared with allogeneic transfusion alone in children undergoing cerebral hematoma evacuation. The secondary objective was to evaluate associated clinical outcomes.

## Materials and methods

### Ethics statement

This study was conducted in accordance with the Declaration of Helsinki. The retrospective cohort design was approved by the Ethics Committee of Beijing Children's Hospital (approval number: IEC-C-006-A04-V.06). The requirement for written informed consent was waived by the institutional review board.

### Study design and patient selection

We retrospectively reviewed medical records of all children who underwent cerebral hematoma evacuation at Beijing Children's Hospital between February 1, 2016, and April 30, 2022. The study classification has been modified to a retrospective cohort study employing propensity score matching. Of 166 consecutive patients identified, 30 were excluded, leaving 136 eligible participants who received intraoperative blood transfusion.

Eligibility criteria included: age 0–18 years; cerebral hematoma evacuation requiring blood transfusion; and availability of complete perioperative laboratory and clinical data. Exclusion criteria were: malignant neoplasm; acute or chronic bacterial or viral infection at admission; congenital malformation; leukemia; hemophilia; or other pre-existing hematological disorders. Patients were allocated to two groups: the ICS group (*n* = 42), receiving combined intraoperative autologous cell salvage and allogeneic blood transfusion; and the ABL group (*n* = 94), receiving allogeneic transfusion only.

### Intraoperative transfusion protocol

Autologous blood was collected using the Cell Saver Elite® (Haemonetics Corporation, Boston, MA, USA). As recommended by the manufacturer, shed blood was mixed with a heparin–saline anticoagulant solution (25,000 IU heparin per 1,000 mL of 0.9% NaCl); this solution was removed during centrifugal washing. Allogeneic red cell and plasma transfusion was administered for hemodynamic instability, anticipated ongoing blood loss, or when the patient-specific minimum acceptable hemoglobin threshold was reached, as determined by the attending anesthesiologist (11). Estimated intraoperative blood loss was calculated as the total volume aspirated into the cell salvage reservoir plus the volume recovered from washed surgical sponges.

Due to the absence of rapid TEG monitoring capability in the operating room and the time-consuming nature of laboratory TEG testing, we did not wait for laboratory test results. The administration of other blood products was generally based on clinical judgment, and this study did not involve any blood products other than plasma.

### Data collection

Baseline characteristics included age, body weight, sex, American Society of Anesthesiologists (ASA) physical status (11), Glasgow Coma Scale (GCS) score (12), and preoperative transfusion status. GCS classification followed established criteria: mild (GCS 13–15); moderate (GCS 9–12, or GCS 13–15 with relevant neuroimaging findings); and severe (GCS ≤8, or GCS 6–12 with intubation, mechanical ventilation, and PICU admission).

Perioperative laboratory measurements—hemoglobin (Hb), hematocrit (Hct), platelet count (PLT), prothrombin time (PT), international normalized ratio (INR), fibrinogen concentration (FIB), and activated partial thromboplastin time (APTT)—were obtained within 24 h before and after surgery. Serum electrolytes (Na+, K+, Ca2+, Cl-) were recorded at both time points.

Clinical outcome measures included operative duration; ICU admission rate; intubation duration; length of hospital stay; hospitalization costs; Glasgow Outcome Scale (GOS) at hospital discharge (13); and postoperative complication rates (epilepsy; surgical site hernia).

Coagulation disorder was defined as any of the following: (a) PLT <100 × 109/L; (b) PT > 16 s or APTT > 45 s (reference upper limits: PT 13.0 s, APTT 35.0 s); or (c) FIB < 1.5 g/L (14).

### Statistical analysis

Sample size adequacy was verified by *post-hoc* power analysis using G*Power 3.1 (two-group *Z*-test; alpha = 0.05, two-tailed). Based on observed coagulation disorder rates (ICS: 8/42; ABL: 41/94), achieved power was 0.85, exceeding the conventional threshold of 0.80. Propensity score matching was performed using multivariate logistic regression with receipt of ICS as the dependent variable and sex, age, body weight, and GCS score as covariates. Nearest-neighbor matching (1:1, without replacement; calipe*r* = 0.02) yielded 41 matched pairs. Post-matching covariate balance was assessed using standardized mean difference (SMD); SMD < 0.10 was considered acceptable.

Continuous variables were assessed for normality using the Kolmogorov–Smirnov test. Normally distributed data are expressed as mean +/- SD and compared by independent-samples *t*-test. Non-normally distributed data are expressed as median (IQR: 25th-75th percentile) and compared by the Mann–Whitney *U*-test. Categorical data are presented as counts and percentages and compared by chi-squared test or Fisher's exact test as appropriate. A two-sided *P*-value < 0.05 was considered statistically significant. All analyses were performed using SPSS version 26.0 (IBM Corp., Armonk, NY, USA).

## Results

### Patient flow and baseline characteristics

Of 166 patients screened, 30 were excluded, yielding 136 eligible participants (ICS: *n* = 42; ABL: *n* = 94). After 1:1 PSM, 41 matched pairs were retained. Before PSM, the groups did not differ significantly in age, body weight, sex distribution, GCS severity category, or preoperative transfusion rate (all *P* > 0.05; Table 1). After PSM, ASA classification remained significantly different between groups (*P* = 0.015), and intraoperative urine output was higher in the ABL group (*P* = 0.012; Table 2). These residual imbalances are acknowledged as limitations.

**Table 1:** Clinical characteristics of pediatric patients before and after propensity score matching.

Variable	Before PSM ICS (*n* = 42)	Before PSM ABL (*n* = 94)		After PSM ICS (*n* = 41)	After PSM ABL (*n* = 41)	*p*-value
Age (years)	6.6 ± 3.9	5.5 ± 3.4	0.150	6.6 ± 4.0	8.1 ± 3.9	0.731
Weight (kg)	26.7 ± 15.1	21.8 ± 15.1	0.089	26.6 ± 15.2	30.0 ± 16.1	0.584
Sex, M:F, n	24:18	56:38	0.790	23:18	22:19	0.824
GCS severity Mild	13 (31.0%)	46 (48.9%)	0.138	13 (31.7%)	22 (53.7%)	0.124
Moderate	6 (14.3%)	16 (17.0%)		6 (14.6%)	5 (12.2%)	
Severe	23 (54.8%)	32 (34.0%)		22 (53.7%)	14 (34.1%)	
Pre−op transfusion, n (%)	2 (4.8%)	6 (6.4%)	0.710	2 (4.8%)	3 (7.3%)	0.644
ASA status II	28 (66.7%)	43 (45.7%)	0.062	28 (68.2%)	17 (41.5%)	0.015
III	9 (21.4%)	27 (28.7%)		8 (19.5%)	8 (19.5%)	
IV	5 (11.9%)	24 (25.5%)		5 (12.2%)	16 (39.0%)	

ICS, intraoperative cell salvage; ABL, allogeneic blood transfusion only; PSM, propensity score matching; GCS, Glasgow Coma Scale; ASA, American Society of Anesthesiologists.

Normally distributed data: mean ± SD; categorical data: *n* (%). *P* < 0.05 considered significant.

**Table 2:** Intraoperative blood product utilization and fluid balance before and after propensity score matching.

Variable	Before PSM ICS (*n* = 42)	Before PSM ABL (*n* = 94)		After PSM ICS (*n* = 41)	After PSM ABL (*n* = 41)	*p*-value
Autologous transfusion (mL)	117 (70, 150)	0	—	117 (70, 154)	0	—
Allogeneic transfusion (mL)	130 (0, 211)	260 (130, 260)	<0.001	130 (0, 228)	260 (130, 390)	<0.001
Total RBC volume (mL)	199 (134, 340)	260 (130, 260)	0.380	199 (134, 341)	260 (130, 390)	0.730
Fresh frozen plasma (mL)	50 (0, 100)	100 (0, 200)	**0.081**	100 (0, 100)	100 (0, 200)	**0.049**
Crystalloid solution (mL)	855 (638, 1363)	650 (475, 1000)	.017	860 (625, 1375)	860 (625, 1375)	0.967
Hydroxyethyl starch (mL)	0 (0, 100)	0 (0, 100)	0.655	0 (0, 100)	0 (0, 100)	0.229
Estimated blood loss (mL)	200 (120, 300)	150 (100, 300)	0.164	200 (120, 300)	200 (100, 300)	0.786
Urine output (mL)	325 (150, 688)	350 (175, 600)	0.912	350 (150, 725)	500 (350, 1000)	0.012

ICS, intraoperative cell salvage; ABL, allogeneic blood transfusion only; PSM, propensity score matching; RBC, red blood cell.

Non-normally distributed data: median (IQR).

*P* < 0.05 significant. Cyan row: plasma utilisation data discussed in Round 2 revision (added to Results and Discussion).

### Blood product utilization

Allogeneic transfusion volume was significantly higher in the ABL group both before PSM (260 [130, 260] vs. 130 [0, 211] mL; *P* < 0.001) and after PSM (260 [130, 390] vs. 130 [0, 228] mL; *P* < 0.001). Total red cell volume (autologous + allogeneic) did not differ significantly before or after PSM (*P* = 0.380 and *P* = 0.730, respectively; Table 2). No adverse transfusion reactions were recorded in either group.

Plasma administration was higher in the ABL group after PSM (100 [0, 200] vs. 100 [0, 100] mL; *P* = 0.049). The volume of plasma transfused should not be used as a diagnostic criterion for coagulation function; the diagnostic criteria for coagulation disorders are described in the Methods section.

### Perioperative laboratory values

Preoperative coagulation indices, electrolytes, Hb, and Hct did not differ significantly between groups before or after PSM (all *P* > 0.05; Table 3). Postoperatively, no significant differences were observed in PT, INR, APTT, PLT, Hb, Hct, or serum electrolytes (all *P* > 0.05; Table 4). Postoperative FIB was numerically higher in the ICS group before PSM (2.2 [1.7, 2.4] vs. 1.8 [1.4, 2.2] g/L; *P* = 0.047), though this difference was not significant after PSM (2.2 [1.7, 2.4] vs. 1.9 [1.5, 2.3] g/L; *P* = 0.110).

**Table 3:** Preoperative coagulation function, electrolyte levels, hemoglobin, and hematocrit before and after propensity score matching.

Parameter	Before PSM ICS (*n* = 42)	Before PSM ABL (*n* = 94)		After PSM ICS (*n* = 41)	After PSM ABL (*n* = 41)	*p*-value
PT (s)	12.40 (12.00, 13.45)	12.90 (12.30, 13.50)	0.083	12.90 (12.23, 13.45)	12.40 (12.00, 13.53)	0.154
NR	1.09 (1.05, 1.18)	1.13 (1.08, 1.18)	0.065	1.13 (1.07, 1.18)	1.09 (1.05, 1.19)	0.150
FIB (g/L)	1.89 ± 0.67	2.07 ± 0.57	0.071	31.85 (29.55, 33.95)	31.35 (28.53,35.25)	0.668
APTT (s)	31.50 (28.65, 34.75)	32.50 (30.40, 35.80)	0.129	32.21 ± 4.42	32.08 ± 4.71	0.905
PLT (x109/L)	294.63 ± 100.57	292.48 ± 72.80	0.844	291.98 ± 87.73	293.39 ± 73.46	0.937
Na + (mmol/L)	136.05 ± 3.29	136.62 ± 3.27	0.355	136.45 (136.70, 138.70)	134.30 (136.30, 138.10)	0.600
K + (mmol/L)	3.94 ± 0.58	3.80 ± 0.49	0.237	3.73 ± 0.53	3.81 ± 0.49	0.488
Ca2+ (mmol/L)	.38 ± 0.16	2.39 ± 0.16	0.205	2.34 ± 0.17	2.39 ± 0.16	0.185
Cl- (mmol/L)	103.04 ± 3.25	103.90 ± 3.27	0.189	103.12 ± 2.96	103.00 ± 3.29	0.873
Hb (g/L)	121.0 (109.5, 126.5)	114.0 (90.0, 123.0)	0.085	121 (115, 130)	121 (103, 127)	0.210
Hct (%)	35.70 (32.10, 36.85)	33.40 (27.10, 36.20)	0.111	36 (34, 38)	35 (31, 37)	0.189

ICS, intraoperative cell salvage; ABL, allogeneic blood transfusion only; PSM, propensity score matching. Normally distributed data: mean ± SD; non-normally distributed: median (IQR). *P* < 0.05 significant. No changes from original submission.

**Table 4:** Postoperative coagulation function, electrolyte levels, hemoglobin, and hematocrit before and after propensity score matching.

Parameter	Before PSM ICS (*n* = 42)	Before PSM ABL (*n* = 94)		After PSM ICS (*n* = 41)	After PSM ABL (*n* = 41)	*p*-value
PT (s)	13.4 (12.9, 14.1)	13.4 (12.9, 14.4)	0.670	13.4 (12.9, 14.1)	13.2 (12.8, 14.3)	0.904
INR	1.16 (1.11, 1.24)	1.20 (1.13, 1.25)	0.737	1.17 (1.12, 1.25)	1.17 (1.12, 1.25)	0.903
FIB (g/L)	2.2 (1.7, 2.4)	1.8 (1.4, 2.2)	0.047	2.2 (1.7, 2.4)	1.9 (1.5, 2.3)	0.110
APTT (s)	32.8 (29.9, 37.9)	33.4 (30.1, 40.4)	0.528	32.8 (29.9, 37.9)	33.2 (30.5, 38.0)	0.600
PLT (x109/L)	215.0 (179.0, 268.0)	219.0 (146.0, 275.8)	0.508	215.0 (179.0, 268.0)	225.0 (163.0, 257.0)	0.553
Na + (mmol/L)	139.41 ± 4.36	139.25 ± 3.85	0.846	139.84 ± 4.25	139.50 ± 4.39	0.734
K + (mmol/L)	3.52 (3.92, 4.11)	3.53 (3.86, 4.10)	0.858	3.85 ± 0.44	3.80 ± 0.47	0.599
Ca2+ (mmol/L)	2.38 ± 0.16	2.39 ± 0.16	0.865	2.30 (2.20, 2.40)	2.22 (2.13, 2.36)	0.252
Cl- (mmol/L)	107.49 ± 4.72	107.82 ± 3.66	0.700	106.60 (104.85, 109.50)	107.25 (104.08, 110.45)	0.757
Hb (g/L)	117.0 (107.0, 129.0)	112.0 (94.5, 126.0)	0.276	120.15 ± 22.43	116.65 ± 19.09	0.453
Hct (%)	33.40 (29.80, 36.50)	31.80 (26.90, 35.90)	0.197	34.15 ± 6.02	33.62 ± 5.43	0.678

ICS, intraoperative cell salvage; ABL, allogeneic blood transfusion only; PSM, propensity score matching. Normally distributed data: mean ± SD; non-normally distributed: median (IQR). *P* < 0.05 significant. Cyan row (FIB): re-validated under Reviewer 2 revised reference limits (PT 13.0 s, APTT 35.0 s); FIB differed before PSM (*P* = 0.047) but not after PSM (*P* = 0.110).

The coagulation function data in Table 4 have been re-validated using the updated reference limits (PT upper limit: 13.0 s; APTT upper limit: 35.0 s). FIB values differed between groups before PSM but this difference was not significant after PSM.

### Coagulation disorders

The composite coagulation disorder rate was significantly lower in the ICS group than the ABL group before PSM (8/42 [19.0%] vs. 42/94 [44.7%]; *P* = 0.008) and after PSM (7/41 [17.1%] vs. 16/41 [39.0%]; *P* = 0.049). Hypofibrinogenemia (FIB < 1.5 g/L) was significantly less frequent in the ICS group before PSM (4/42 [9.5%] vs. 29/94 [30.9%]; *P* = 0.014) and after PSM (3/41 [7.3%] vs. 11/41 [26.8%]; *P* = 0.040). No significant differences were found in thrombocytopenia, PT prolongation, or APTT prolongation between groups before or after PSM (Table 5).

**Table 5:** Clinical outcomes and postoperative coagulation disorders before and after propensity score matching.

Outcome	Before PSM ICS (*n* = 42)	Before PSM ABL (*n* = 94)		After PSM ICS (*n* = 41)	After PSM ABL (*n* = 41)	*p*-value
Operating time (min)	162 (133, 196)	140 (110, 187)	0.034	175 (140, 208)	164 (138, 195)	0.743
ICU admission, n (%)	28 (66.7%)	73 (77.7%)	0.175	27 (65.9%)	34 (83.0%)	0.077
Intubation duration (h)	2 (0, 12)	2 (1, 21)	0.387	2 (0, 13)	2 (1, 29)	0.335
Hospital stay (days)	10 (5, 16)	8 (5, 12)	0.072	11 (6, 17)	8 (5, 13)	0.175
Post−op transfusion, n (%)	7 (16.7%)	33 (36.1%)	0.029	7 (17.1%)	11 (26.8%)	0.286
Epilepsy	5 (11.9%)	9 (9.6%)	0.679	5 (12.1%)	7 (17.1%)	0.532
Hernia	18 (42.9%)	47 (50.0%)	0.441	17 (41.5%)	26 (63.4%)	0.047
Any coagulation disorder	8/42 (19.0%)	42/94 (44.7%)	0.008	7/41 (17.1%)	16/41 (39.0%)	0.049
PLT <100 × 10^9^/L	1/42 (2.4%)	11/94 (11.7%)	0.149	1/41 (2.4%)	3/41 (7.3%)	0.608
**PT** **>** **16 s (ref: 13.0 s)**	**1/42 (2.4%)**	**3/94 (3.2%)**	**1.000**	**1/41 (2.4%)**	**2/41 (4.9%)**	**1.000**
**APTT > 45 s (ref: 35.0 s)**	**3/42 (7.1%)**	**11/94 (11.7%)**	**0.615**	**3/41 (7.3%)**	**5/41 (12.2%)**	**0.710**
FIB < 1.5 g/L	4/42 (9.5%)	29/94 (30.9%)	0.014	3/41 (7.3%)	11/41 (26.8%)	0.040
GOS 1 – Death	2 (4.8%)	4 (4.3%)	0.987	2 (4.8%)	4 (9.8%)	0.012
GOS 2 – Vegetative	1 (2.4%)	4 (4.3%)		1 (2.4%)	4 (9.8%)	
GOS 3 – Severe disability	6 (14.3%)	14 (14.9%)		5 (12.2%)	12 (29.3%)	
GOS 4 – Mild disability	5 (11.9%)	10 (10.6%)		5 (12.2%)	9 (22.0%)	
GOS 5 – Good recovery	28 (66.7%)	62 (66.0%)		28 (68.3%)	12 (29.3%)	
Hospitalization cost (CNY)	47,475 (38,821, 63,768)	61,547 (42,047, 76,848)	0.059	53,420 (40,874, 76,603)	63,130 (42,010, 77,250)	0.495

ICS, intraoperative cell salvage; ABL, allogeneic blood transfusion only; PSM, propensity score matching; ICU, intensive care unit; GOS, Glasgow Outcome Scale (1 = death, 2 = vegetative, 3 = severe disability, 4 = mild disability, 5 = good recovery); PLT, platelet count; PT, prothrombin time; APTT, activated partial thromboplastin time; FIB, fibrinogen; CNY, Chinese yuan. Non-normally distributed data: median (IQR); categorical: *n* (%). *P* < 0.05 significant. Cyan rows: PT and APTT threshold labels updated per Reviewer 2 (PT > 16 s, APTT > 45 s; reference upper limits PT 13.0 s, APTT 35.0 s)^.^

### Clinical outcomes

Before PSM, operating time was longer in the ICS group (162 [133, 196] vs. 140 [110, 187] min; *P* = 0.034) and the postoperative transfusion rate was higher in the ABL group (36.1% vs. 16.7%; *P* = 0.029). After PSM, neither difference remained significant (*P* = 0.743 and *P* = 0.286). ICU admission, intubation duration, hospital stay, epilepsy rate, and hospitalization costs did not differ significantly between groups before or after PSM (all *P* > 0.05; Table 5). The postoperative hernia rate was significantly higher in the ABL group after PSM (63.4% vs. 41.5%; *P* = 0.047); given the multifactorial etiology of this complication, this result is interpreted cautiously.

GOS distribution was similar before PSM (*P* = 0.987); after PSM, GOS distribution was significantly different (*P* = 0.012), with the ICS group showing a higher proportion of good recovery (68.3% vs. 29.3%). This finding must be interpreted cautiously given the small matched sample size.

## Discussion

This retrospective propensity score-matched cohort study examined the hemostatic and clinical consequences of combining intraoperative cell salvage with allogeneic transfusion vs. allogeneic transfusion alone in children undergoing cerebral hematoma evacuation. The principal findings were: (i) combined ICS + allogeneic transfusion significantly reduced allogeneic blood consumption without altering total red cell volume; (ii) the composite coagulation disorder rate—particularly hypofibrinogenemia—was significantly lower in the ICS group both before and after PSM; (iii) standard postoperative coagulation indices, electrolytes, Hb, and Hct did not differ significantly; and (iv) clinical outcomes were broadly comparable after PSM, with an exploratory GOS advantage in the ICS group requiring prospective confirmation.

### ICS does not impair postoperative coagulation

A theoretical concern regarding ICS in neurosurgical patients is that reinfusion of shed blood containing activated coagulation factors and fibrin degradation products may exacerbate systemic coagulopathy. The present data do not support this concern. No significant differences were detected between groups in PT, APTT, INR, or PLT after surgery, consistent with prior reports demonstrating that centrifugal washing removes most procoagulant mediators (7, 8). Previous reports demonstrated that administering salvaged blood in quantities exceeding an entire blood volume may not result in coagulopathy, provided an appropriate amount of non-RBC blood components is transfused concurrently (15). Intraoperative cell salvage and retransfusion in small children with complex congenital heart defects undergoing cardiopulmonary bypass resulted in significantly fewer donor exposures without an increase in postoperative bleeding (16).

Importantly, the composite coagulation disorder rate—and specifically hypofibrinogenemia (FIB < 1.5 g/L)—was significantly lower in the ICS group both before and after PSM. The lower allogeneic transfusion volume in the ICS group likely reduced the dilutional and immunomodulatory burden on the coagulation cascade (17, 18), leading to better-preserved fibrinogen levels. In infants and young children, ICS application has been limited by the difficulty of collecting and efficiently processing the small volumes of blood lost during surgery (19).

### ICS reduces allogeneic blood requirements

The consumption of allogeneic RBCs was significantly lower in the ICS group, while total red cell volume did not differ, confirming that autologous reinfusion functionally offset a proportion of allogeneic exposure. This aligns with previous literature (20, 21). Reducing allogeneic exposure is particularly important in children, as the three primary causes of transfusion-associated mortality—TRALI, TACO, and hemolytic transfusion reactions—carry reported mortality rates of 15%–30% (22). Maintaining normovolemia is especially critical in children undergoing craniotomy, as hypovolemia represents the most common identifiable cause of anesthesia-related cardiac arrest in the pediatric population (23).

Notably, plasma administration was significantly higher in the ABL group after PSM (*P* = 0.049), suggesting greater reliance on allogeneic blood products in that group. However, the volume of plasma transfused should not be interpreted as a surrogate marker for coagulation function status, as the diagnostic criteria for coagulation disorders have been explicitly defined in the Methods section.

### Effect of ICS on clinical outcomes

After PSM, no significant differences were detected in ICU admission, intubation duration, hospital stay, epilepsy rate, or hospitalization costs, demonstrating that the addition of ICS did not increase perioperative burden. The preoperative difference in operating time was no longer significant after matching, suggesting it reflected case complexity rather than ICS-related procedural delay. The postoperative hernia rate was significantly higher in the ABL group after PSM (*P* = 0.047); however, surgical site hernia following craniotomy is multifactorial and is unlikely to be mechanistically explained by transfusion type alone.

The GOS distribution after PSM was statistically significant (*P* = 0.012), with the ICS group showing higher rates of good recovery (68.3% vs. 29.3%) and lower mortality (4.8% vs. 9.8%). While this is a notable signal, several important caveats apply: the matched sample is small (*n* = 41 per group); GOS was assessed at hospital discharge; and the retrospective design does not permit causal inference. It is plausible that the significantly lower coagulation disorder rate—particularly reduced hypofibrinogenemia—contributed to more favorable neurological outcomes, as TBI-associated coagulopathy has been independently linked to worse prognosis (17, 18). This hypothesis should be tested prospectively.

### Electrolyte safety

No significant differences in serum Na+, K+, Ca2+, or Cl- were observed between groups before or after PSM. This indicates that combined autologous and allogeneic transfusion does not induce clinically significant electrolyte imbalance in this pediatric cohort, including hyperkalemia—a recognized risk with large-volume stored allogeneic blood.

### Limitations

Several limitations warrant consideration. First, the retrospective single-center design precluded standardization of transfusion triggers and blood product administration protocols. Second, the total cohort and matched sample sizes were small, limiting power to detect modest between-group differences. Third, PSM did not fully balance ASA classification and intraoperative urine output, leaving residual confounding.

Since the decision to administer blood products is primarily made by anesthesiologists upon entering the operating room, the ASA classification serves as an indication of the patient's condition at that moment. It is precisely because clinical anesthesiologists determine that the pediatric patient's condition is worse that the patient is more likely to receive allogeneic blood transfusion first, in order to ensure a smooth transition of the circulatory status. Conversely, if the pediatric patient's condition is stable and there is no urgent need for blood transfusion, allogeneic blood transfusion should not be administered first. Instead, intraoperative monitoring can be conducted, with blood products administered only when required.

Fourth, autologous-to-allogeneic volume ratios were not analyzed, preventing identification of a threshold below which partial ICS ceases to be hemostasis-preserving. Fifth, standard coagulation tests were used exclusively; viscoelastic hemostatic assays (TEG or ROTEM), which provide superior characterization of the complete hemostatic profile in TBI, were not routinely employed (24). Sixth, GOS was assessed at hospital discharge rather than at standardized longer-term follow-up intervals (e.g., 3 or 6 months). Finally, single-institution results may have limited generalizability. A prospective multicenter randomized controlled trial incorporating viscoelastic hemostatic monitoring and standardized long-term neurological follow-up is necessary to generate definitive evidence.

## Conclusion

In children undergoing cerebral hematoma evacuation following traumatic brain injury, combined intraoperative cell salvage plus allogeneic transfusion was associated with significantly reduced allogeneic blood consumption and a lower incidence of postoperative coagulation disorders—particularly hypofibrinogenemia—compared with allogeneic transfusion alone, both before and after propensity score matching. Standard postoperative coagulation indices and electrolyte balance were not significantly impaired. An exploratory advantage in GOS distribution was observed after PSM; this finding requires prospective confirmation. Partial intraoperative autologous blood salvage represents a safe, hemostasis-preserving adjunct to blood management in pediatric neurosurgical trauma.

## Data Availability

The original contributions presented in the study are included in the article/Supplementary Material, further inquiries can be directed to the corresponding author.
